# Imbalance of Homocysteine and H_2_S: Significance, Mechanisms, and Therapeutic Promise in Vascular Injury

**DOI:** 10.1155/2019/7629673

**Published:** 2019-11-22

**Authors:** Qin Yang, Guo-Wei He

**Affiliations:** ^1^Center for Basic Medical Research & Department of Cardiovascular Surgery, TEDA International Cardiovascular Hospital, Chinese Academy of Medical Sciences & Peking Union Medical College, Tianjin, China; ^2^School of Pharmacy, Wannan Medical College, Wuhu, Anhui, China; ^3^Department of Surgery, Oregon Health and Science University, Portland, Oregon, USA

## Abstract

While the role of hyperhomocysteinemia in cardiovascular pathogenesis continuously draws attention, deficiency of hydrogen sulfide (H_2_S) has been growingly implicated in cardiovascular diseases. Generation of H_2_S is closely associated with the metabolism of homocysteine via key enzymes such as cystathionine *β*-synthase (CBS) and cystathionine *γ*-lyase (CSE). The level of homocysteine and H_2_S is regulated by each other. Metabolic switch in the activity of CBS and CSE may occur with a resultant operating preference change of these enzymes in homocysteine and H_2_S metabolism. This paper presented an overview regarding (1) linkage between the metabolism of homocysteine and H_2_S, (2) mutual regulation of homocysteine and H_2_S, (3) imbalance of homocysteine and H_2_S in cardiovascular disorders, (4) mechanisms underlying the protective effect of H_2_S against homocysteine-induced vascular injury, and (5) the current status of homocysteine-lowering and H_2_S-based therapies for cardiovascular disease. The metabolic imbalance of homocysteine and H_2_S renders H_2_S/homocysteine ratio a potentially reliable biomarker for cardiovascular disease and development of drugs or interventions targeting the interplay between homocysteine and H_2_S to maintain the endogenous balance of these two molecules may hold an even bigger promise for management of vascular disorders than targeting homocysteine or H_2_S alone.

## 1. Association between H_2_S Generation and Homocysteine Metabolism

### 1.1. Biosynthesis and Catabolism of Homocysteine

Homocysteine is a nonproteinogenic, sulfur-containing amino acid formed during metabolism of the essential amino acid methionine. Plasma level of homocysteine is determined by a balance between its biosynthesis and catabolism, which in healthy subjects is below 15 *μ*mol/L. Synthesis of homocysteine via transmethylation of methionine is catalyzed by enzymes namely S-adenosylmethionine (SAM) synthetase, methyltransferase (MT), and S-adenosylhomocysteine (SAH) hydrolase in three sequential steps: formation of SAM by SAM synthetase-catalyzed reaction of methionine and ATP, conversion of SAM to SAH by methyl transfer reaction catalyzed by MT, and SAH metabolized to adenosine and homocysteine by SAH hydrolase [1, 2].

Homocysteine is catabolized by two means: remethylation to methionine and transsulfuration to cysteine. Remethylation of homocysteine involves folate/vitamin B12-dependent and vitamin B12-independent mechanisms. The former uses N-5-methyl tetrahydrofolate (THF) as a methyl group donor under catalysis of the vitamin B12-dependent enzyme methionine synthase (MS), while the later relies on the methyl group donated by betaine and requires betaine-homocysteine S-MT for catalysis. Transsulfuration of homocysteine is catalyzed by the vitamin B6-dependent enzymes: cystathionine *β*-synthase (CBS) and cystathionine *γ*-lyase (CSE). CBS converts homocysteine and serine into cystathionine, which is taken up by CSE to generate cysteine [1, 2]. CBS and CSE are also the major enzymes responsible for the biogenesis of hydrogen sulfide (H_2_S), a gasotransmitter known for its regulatory role in many physiological processes [3]. A small portion of homocysteine, approximately 5~10% of the total daily cellular production that is not metabolized within the cell, is exported to the plasma compartment and such baseline value is maintained in healthy human subjects by a constant clearance through the kidney [4].

### 1.2. H_2_S Biogenesis and Catabolism

**Figure 1:**
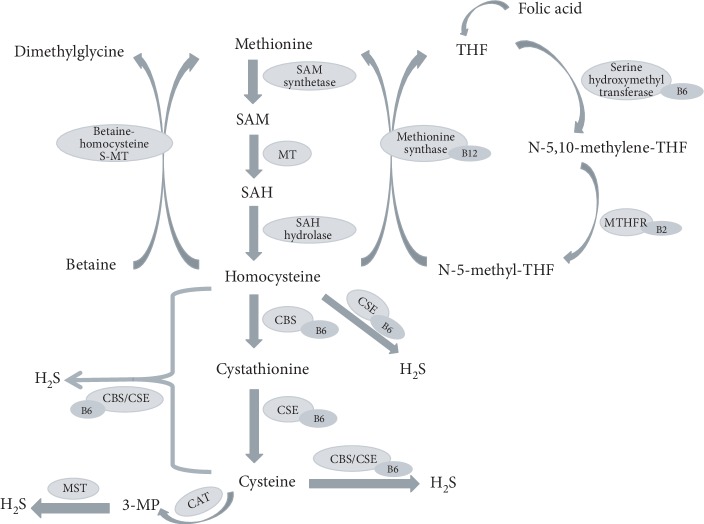
Schematic overview of the association between homocysteine and H_2_S. Homocysteine is biosynthesized from methionine by S-adenosylmethionine (SAM) synthetase, methyltransferase (MT), and S-adenosylhomocysteine (SAH) hydrolase in sequential steps. Homocysteine can be either remethylated to methionine through folate/vitamin B12-dependent or vitamin B12-independent mechanisms, or transsulfurated to cysteine under the catalysis of cystathionine *β*-synthase (CBS) and cystathionine *γ*-lyase (CSE) that requires vitamin B6 as an enzyme cofactor. Homocysteine and cysteine are substrates for H_2_S production, and the generation of H_2_S is catalyzed by CBS, CSE, and 3-mercaptopyruvate sulfurtransferase (MST). THF: tetrahydrofolate; 3-MP: 3-mercaptopyruvate; CAT: cysteine aminotransferase; MTHFR: N-5,10-methylenetetrahydrofolate reductase.

H_2_S is endogenously generated in mammalian tissues via independent reactions catalyzed by CBS, CSE, and 3-mercaptopyruvate sulfurtransferase (MST) [5, 6]. CBS produces H_2_S from cysteine via a *β*-elimination reaction, and CSE generates H_2_S via *α*,*β*-elimination of cysteine (cysteine + H_2_O ⟶ serine + H_2_S). Both CBS and CSE can catalyze *β*-replacement reaction, which condenses two cysteine molecules, or catalyze the condensation reaction of homocysteine with cysteine through *β*- or *γ*-replacement, to produce H_2_S [7] (cysteine + cysteine ⟶ lanthionine + H_2_S; cysteine + homocysteine ⟶ cystathionine + H_2_S). In addition, both CBS and CSE can catalyze cysteine *α*,*β*-elimination to yield cysteine persulfide that ultimately may generate H_2_S [7] (cysteine ⟶ cysteine persulfide + pyruvate + NH_3_ ⟶ H_2_S). It was reported that human CBS is much more active at producing H_2_S by a *β*-replacement reaction than by a *β*-elimination reaction [8]. CSE alone may produce H_2_S via homocysteine *α*, *γ*-elimination (homocysteine + H_2_O ⟶ homoserine + H_2_S ⟶ *α*-ketobutyrate + NH_3_) or *γ*-replacement (homocysteine + homocysteine ⟶ homolanthionine + H_2_S) [9]. MST is another H_2_S-generating enzyme that is tightly associated with cysteine metabolism. It acts in conjunction with cysteine aminotransferase (CAT) to produce H_2_S. MST produces H_2_S from 3-mercaptopyruvate (3-MP), which is generated by CAT from cysteine in the presence of *α*-ketoglutarate [10, 11] (cysteine + *α*-ketoglutarate ⟶ 3-MP + glutamate, 3-MP + MST ⟶ MST persulfide intermediate + pyruvate, MST persulfide intermediate + thiol-containing substrates (RSH) or reduced thioredoxin ⟶ disulfide (RSSR) or oxidized thioredoxin + H_2_S). A recent study provided evidence suggesting that in addition to L-cysteine, D-cysteine also serves a substrate for 3-MP generation thereof H_2_S production, particularly in the cerebellum and kidney [12].  Figure 1 schematically describes the association between H_2_S generation and homocysteine metabolism.

Although the expression of CBS, CSE, and MST shows tissue-specific dominance, i.e., CBS and MST predominate in the brain and kidney and CSE abundantly exists in the liver and in vascular and nonvascular smooth muscle, these H_2_S-producing enzymes are generally ubiquitously expressed in mammalian tissues with H_2_S produced to impact a wide range of cellular processes [13]. All three enzymes are found to be expressed in vascular endothelial and smooth muscle cells [14, 15], which is the biochemical basis underlying the pathophysiological significance of H_2_S in vasculatures.

## 2. Influence of Hyperhomocysteinemia on H_2_S Metabolism

Hyperhomocysteinemia occurs as a result of increased synthesis and/or decreased catabolism (remethylation and transsulfuration) of homocysteine. In recognition of severity and pathogenic mechanisms, hyperhomocysteinemia is categorized into three classes as mild, moderate, and severe hyperhomocysteinemia with plasma homocysteine level ranging from 15 to 30 *μ*mol/L, 31 to 100 *μ*mol/L, and >100 *μ*mol/L, respectively [16]. Nutritional deficiency of folic acid and vitamin B6 and/or B12 and renal insufficiency often cause relatively mild hyperhomocysteinemia, while genetic disorders such as mutations in N-5,10-methylenetetrahydrofolate reductase (MTHFR) and CBS may result in moderate and severe hyperhomocysteinemia [17, 18].

H_2_S biosynthesis is affected by hyperhomocysteinemia. Under normal conditions, plasma total cysteine in humans (∼250 *μ*mol/L) is much higher than the concentration of total homocysteine (∼6-15 *μ*mol/L) [19]. Chiku and colleagues reported that at physiological concentrations of homocysteine and cysteine, approximately 70% of H_2_S is produced from cysteine through CBS-/CSE-catalyzed *α*,*β*-elimination of cysteine. However, in the state of homocysteine accumulation, homocysteine substitutes cysteine becoming a significant source of H_2_S in moderate and the principal source of H_2_S in severe hyperhomocysteinemia. As a result, H_2_S generated by CSE through the *α*,*γ*-elimination and *γ*-replacement reactions of homocysteine is dramatically enhanced [9]. In contrast to CSE, CBS-catalyzed H_2_S-generating reactions are insensitive to the grade of hyperhomocysteinemia [20]. Such difference suggests that CSE may be primarily responsible for H_2_S production change under conditions of hyperhomocysteinemia.

It was reported that increase of homocysteine causes a decrease of H_2_S production. For example, plasma H_2_S level was found to be lowered in hyperhomocysteinemic mice [21], and intracerebroventricular injection of homocysteine in rats resulted in decreased generation of endogenous H_2_S in the hippocampus [22]. Reduction of H_2_S was also observed in cells exposed to homocysteine [21, 23]. The decrease of H_2_S was attributed to suppressed expression/activity of the H_2_S-generating enzymes CBS [22, 23] and CSE [21]. Further studies demonstrated that homocysteine-induced transcriptional repression of CSE in macrophages is a result of increased DNA methyltransferase expression and DNA hypermethylation in CSE promoter region [21]. Homocysteine was also found to capture H_2_S anion to form homocysteine persulfide [24], which weakened the cardioprotective effect of H_2_S in hyperhomocysteinemic animals subjected to ischemia-reperfusion injury.

Despite accumulating evidence in support of the inhibitory effect of homocysteine on H_2_S generation, there were anomalous reports of elevated H_2_S levels in hyperhomocysteinemia. Compared with healthy volunteers, hyperhomocysteinemic patients with MTHFR C677T polymorphism showed a significantly higher content of H_2_S in either platelets or plasma [25]. Similarly, in hyperhomocysteinemic mice bearing MTHFR or CBS mutations, H_2_S level in retinas was markedly increased, as compared to wild-type animals [26]. The mechanisms underlying the elevated H_2_S level in these studies remain elusive, though the investigators ruled out the possibility of upregulation of CSE and MST [26].

## 3. Regulation of Homocysteine Metabolism by H_2_S

On the one hand, homocysteine regulates H_2_S production; on the other hand, the level of homocysteine is regulated by H_2_S. It was observed that intraperitoneal injection of H_2_S gas saturation solution significantly reduces plasma total homocysteine concentration in hyperhomocysteinemic rat induced by subcutaneous injection of homocysteine [27]. Treating myoblasts with the H_2_S donor sodium hydrosulfide (NaHS) significantly lowered homocysteine content in the cell. Conversely, deficient endogenous H_2_S production induced by CSE siRNA was concomitant with the increased homocysteine level [28]. Oral administration of a new H_2_S-releasing compound ACS94 to healthy rats increases the concentration of circulating H_2_S and decreases homocysteine level in plasma and organs [29]. In an in vitro study of mouse brain endothelial cells, Tyagi and colleagues found that NaHS decreases homocysteine accumulation in cells exposed to high concentration of methionine, concluding that H_2_S is a potent inhibitor of homocysteine formation [30].

In a recent study of the antihypertrophic effect of H_2_S against homocysteine on cardiomyocytes, Nandi and associates observed differential effects of H_2_S and homocysteine on the expression of CBS and CSE along with a finding of a negative feedback regulation between these two enzymes [31]. Elevated levels of homocysteine downregulated CBS but upregulated CSE whereas H_2_S downregulated CSE but upregulated CBS in cardiomyocytes, indicating the negative feedback between CBS and CSE, which can be influenced by hyperhomocysteinemia or H_2_S. The direct regulation of CSE by CBS was further confirmed in CBS-deficient hyperhomocysteinemic animals. As compared to CBS^+/+^ siblings, CBS^+/−^ mice exhibit upregulated CSE in the heart, suggesting that CBS deficiency induces CSE. Further mechanistic exploration revealed that homocysteine-induced CBS deficiency enhances the activity of specificity protein-1 (SP1), an inducer for CSE, and downregulates miR-133, a SP-1 targeting microRNA. On the contrary, H_2_S suppresses CSE by inhibiting SP1 directly and also indirectly by inducing miR-133a, which consequently leads to CBS upregulation.

## 4. Imbalance of Homocysteine and H_2_S in Cardiovascular Disease

Considering both evidence of defective and enhanced H_2_S production under hyperhomocysteinemic conditions and the interplay between homocysteine and H_2_S, the change of H_2_S/homocysteine ratio may be more valuable than the absolute concentration change of H_2_S and homocysteine in depicting the role of these metabolites in disease pathogenesis. Hypertensive children in comparison to normotensive children showed significantly lower plasma H_2_S/homocysteine ratio due to increased homocysteine concentration and decreased H_2_S level, and a negative correlation existed between systolic blood pressure and the plasma H_2_S/homocysteine ratio [32]. Similarly, decreased levels of H_2_S and increased levels of homocysteine were shown to be significantly negatively correlated in pulmonary hypertension associated with congenital heart disease, which was attributed to decreased expressions of MTHFR and CSE along with vitamin B12 deficiency [33]. He and colleagues found that although patients with chronic obstructive pulmonary disease (COPD) and concomitant cardiovascular disease (CVD) have higher H_2_S and homocysteine levels than those without CVD but only COPD, the H_2_S/homocysteine ratio in serum from COPD + CVD patients was significantly lower than that from the COPD group, and such ratio was positively correlated with lung function [34]. These studies supported the notion of metabolic imbalance of homocysteine and H_2_S in cardiovascular pathologies and as comparing to homocysteine or H_2_S alone, the ratio of H_2_S to homocysteine may be a more reliable biomarker to predict risk of cardiovascular disease.

## 5. Role of Hyperhomocysteinemia and H_2_S Deficiency in Vascular Pathologies

Hyperhomocysteinemia is recognized as an independent risk factor for cardiovascular, cerebrovascular, and peripheral artery disease and its association with atherosclerosis, hypertension, coronary artery disease, stroke, *etc.* has been well-documented [35]. For example, in patients with coronary artery disease, the plasma homocysteine level was revealed as a strong predictor of cardiovascular mortality [36], and the severity of atherosclerosis was demonstrated to be correlated with the plasma level of homocysteine [37]. Also, evidence in support of the role of H_2_S deficiency in vascular disorders such as hypertension and atherosclerosis keeps growing [38, 39]. A couple of newly published review articles addressed vascular biology of H_2_S [40] and the mechanisms of vascular injury induced by hyperhomocysteinemia [41]. The role of oxidative stress, endoplasmic reticulum (ER) stress, and regulation of DNA methylation in homocysteine-induced endothelial dysfunction and vascular inflammation has been discussed in depth [41]. In light of the linkage between homocysteine and H_2_S, the focus of the ensuing sections is to discuss the underlying basis of vasoprotection conferred by H_2_S against hyperhomocysteinemia.

## 6. H_2_S Antagonizes Homocysteine-Induced Vascular Injury: Role of NO Signaling

Conversion of homocysteine to H_2_S has been found to improve renovascular function in hyperhomocysteinemia. In the presence of a high level of homocysteine, compared with nontransfected renal arteries, arteries transfected with CBS, CSE, and MST triple genes generated more H_2_S and were more responsive to endothelium-dependent vasodilator, accompanied by an increased expression of eNOS protein [42]. Reduced caveolin-1 expression also contributes to increased eNOS activity, as demonstrated in CBS^+/−^ hyperhomocysteinemic mice receiving H_2_S treatment who showed attenuation in renovascular smooth muscle cell proliferation and decrease in blood pressure [43]. Previous in vitro and in vivo studies of hyperhomocysteinemia have attributed the inhibition of eNOS and the consequent reduction of nitric oxide (NO) bioavailability to homocysteine-induced eNOS gene inhibition, eNOS inactivation (decreased phosphorylation at activating site and increased phosphorylation at inhibitory site), eNOS uncoupling, and arginase activation [44–47]. Szabo in his recent review article summarized the mechanisms by which H_2_S enhances eNOS-NO signaling, including increasing eNOS mRNA synthesis, stimulating eNOS activity via Ca^2+^ mobilization and Akt-mediated phosphorylation, direct sulfhydration of eNOS, and maintaining soluble guanylate cyclase (sGC) in an NO-activatable state, reacting with cGMP to yield PDE5-resistant 8-SH-cGMP and inhibiting PDE5 activity [48]. Out of a pile of evidence suggesting H_2_S-induced stimulation of eNOS-NO, there is rebuttal evidence. In a latest study using CSE^–/–^ mice, Szijártó and colleagues demonstrated that lack of CSE-produced H_2_S is associated with higher NO bioavailability in peripheral arteries. They attributed this to a decrease in NO scavenging, which occurs through direct interaction of H_2_S and NO resulting in nitroxyl (HNO) formation [49].

Not only H_2_S regulates NO production/activity, NO also influences H_2_S-induced response. eNOS^−/−^ mice exhibited significantly enhanced relaxation to L-cysteine in carotid arteries whereas overexpression of eNOS suppressed L-cysteine-induced relaxation, which suggested that endogenously produced H_2_S can compensate for impaired vasodilatory responses when NO is deficient while eNOS/NO abundance limits endogenous H_2_S-induced vascular responses [50]. The cross-talk between H_2_S and NO in different grades of hyperhomocysteinemia is worthy of study, which will help us gain a comprehensive understanding of the role of these two important gasotransmitters in vascular pathology associated with hyperhomocysteinemia.

## 7. H_2_S Antagonizes Homocysteine-Induced Vascular Injury: Role of Oxidative Stress

Oxidative stress has been strongly implicated in vascular injury and remodeling in hyperhomocysteinemia. Homocysteine induces oxidative stress through multiple mechanisms, including (1) homocysteine autooxidation. When homocysteine binds via a disulfide bridge with plasma proteins (mainly albumin), or other low-molecular plasma thiols, or a second homocysteine molecule, autooxidation of the free thiol group of homocysteine occurs, leading to generation of hydrogen peroxide (H_2_O_2_) and reactive radical oxygen species, superoxide and hydroxyl radical; (2) imbalance between oxidant and antioxidant enzymes, e.g., activation of NADPH oxidases and inhibition of superoxide dismutase (SOD); (3) eNOS-dependent generation of superoxide anion via eNOS uncoupling, which may be triggered by homocysteine-induced decrease of tetrahydrobiopterin (BH4) [45, 51, 52].

H_2_S inhibits homocysteine-induced oxidative stress. In vitro cell culture experiments demonstrated that H_2_S precursor NaHS effectively lowered reactive oxygen/nitrogen species (ROS/RNS) production and normalized redox enzyme levels in vascular smooth muscle and endothelial cells subjected to homocysteine exposure [30, 53]. Further investigation revealed the contributing role of mitochondria in homocysteine-induced ROS production and imbalance of NOX-4 and SOD-2, and the antioxidative and antimitotoxic properties of H_2_S in mediating endothelial protection [54]. In mice which accepted intracerebral injection of homocysteine, treatment with NaHS significantly ameliorated cerebrovascular dysfunction and neurodegeneration. The protection was associated with suppressed oxidative stress, indicated by decreased malondialdehyde and increased glutathione [55]. Similar results were also obtained in hyperhomocysteinemic mice orally taking homocysteine. Restoration of plasma H_2_S level by H_2_S supplementation ameliorated homocysteine-induced neurovascular remodeling with concomitant decreases in superoxide and nitrite and increases in SOD, catalase, glutathione, *etc.* [56] The ability of H_2_S in enhancing the activity of *γ*-glutamylcysteine synthetase, the committing step in the synthesis of glutathione; and upregulating transport of cysteine, the rate-limiting substrate of glutathione synthesis [57] may provide an explanation for the increased level of this antioxidant in hyperhomocysteinemic mice treated with NaHS. A recent study suggested that H_2_S may also function as a heme-redox-intermediate-scavenging antioxidant [58]. H_2_S mitigates hemoglobin oxidation which thereby inhibits oxidized hemoglobin-induced lipid peroxidation and the consequent atherosclerotic lesion in the vessel wall in both human and mouse. Such reduction of hemoglobin oxidation species may take part in the cytoprotective effects conferred by H_2_S against homocysteine since homocysteine has been shown to enhance hemoglobin oxidation [59].

It has been well-known that oxidative stress can trigger inflammatory responses and these two constitute a mutual reinforcing system in the development of atherosclerosis. By focusing on matrix metalloproteinases (MMPs) in atherosclerosis, Vacek and coworkers reviewed the evidence that H_2_S may deactivate homocysteine-induced MMP activities, resulting in a decrease of smooth muscle proliferation and suppression of vascular inflammation and remodeling [60]. The finding of alleviation of inflammatory responses via antioxidant-dependent inhibition of MMPs enriched our understanding of the vascular protection conferred by H_2_S in hyperhomocysteinemia.

## 8. Role of Subcellular Machineries in Homocysteine-Induced Oxidative Stress: Effect of H_2_S

### 8.1. Mitochondrion

Being a major source of endogenous ROS, mitochondria significantly contributes to excessive ROS generation induced by homocysteine and have been shown to be an essential target for H_2_S. In studies of mouse brain endothelial cells (bEnd3), Kamat and colleagues demonstrated that homocysteine induces upregulation of N-methyl D-aspartate (NMDA-R1), a receptor for homocysteine by increasing DNA methylation, leading to NOX-4 overexpression and mitochondrial superoxide production. NaHS treatment downregulated NMDA-R1 expression, maintained mitochondrial integrity, and attenuated mitochondrial redox stress caused by homocysteine. Amelioration of mitochondrial toxicity by NaHS through antagonizing NMDA-R1 protects the integrity and function of endothelial cells, shown by preserved eNOS and endothelin-1 expression. Moreover, experiments through modulating CSE further demonstrated the role of endogenous H_2_S in inhibiting mitochondrial superoxide generation and mitochondrial toxicity [54]. In vitro studies of isolated mitochondria from mouse aortic endothelial cells showed that homocysteine increased ROS production, particularly H_2_O_2_, in the mitochondria, which was associated with increased mitophagy. Gene delivery of CBS, CSE, or MST to the cells inhibited ROS production and mitigated mitophagy [42].

### 8.2. Endoplasmic Reticulum

While prone to oxidative damages, endoplasmic reticulum (ER) is also a ROS generator. As characterized by an elevated ratio of oxidized to reduced glutathione (GSSG/GSH), the lumen of ER is an oxidizing environment enriched with protein disulfide isomerase (PDI) and ER oxidoreductase (ERO)-1*α*, which allows the proper native disulfide bond formation and the resultant proper protein folding (polypeptide rearrangement to reach the native conformational state of the protein). During disulfide bond-dependent protein folding, electrons are transferred from the target cysteine residues to molecular oxygen, generating H_2_O_2_; however, in stressed ER, nonnative disulfide bond formation is increased, resulting in GSH consumption as a protective mechanism, which leads to GSH depletion contributing to excessive ROS generation and consequent development of oxidative stress [61]. The role of ER stress and the cross-talk between ER stress and oxidative stress in mediating endothelial dysfunction have been suggested in several pathological conditions including homocysteine exposure [62–65]. There are several lines of evidence suggesting that H_2_S could prevent homocysteine-induced ER stress [66–69], though these results were not derived from studies of vessels but from the skeletal muscle, neural system, and cardiomyocytes, it is very likely that the anti-ER stress capacity of H_2_S is also involved in its protective effect on vasculatures in hyperhomocysteinemic conditions, which however warrants further investigation.

In a recent study, Kabil and colleagues demonstrated that ER stress induces CSE and causes inhibition of CBS by binding with CO, a product of heme oxygenase-1 in response to ER stress, leading to a build-up of homocysteine and a decrease in cystathionine, which combined to flip the operating preference of CSE from cystathionine to cysteine thus favors the production of H_2_S [70]. As homocysteine is known to act as an ER stress inducer, the metabolic switch in the activity of transsulfuration pathway enzymes in response to ER stress and the consequent increase of H_2_S synthesis may serve as an endogenous cardioprotective mechanism in hyperhomocysteinemia.

We recently demonstrated that ER stress mediates homocysteine-induced vascular dysfunction via suppressing calcium-activated potassium (K_Ca_) channels [71–73]. ER stress inhibited the cell surface expression of intermediate and small conductance K_Ca_ (IK_Ca_ and SK_Ca_) channels in endothelium [71] and enhanced the ubiquitin ligase-mediated loss of the *β*1 subunit of large conductance K_Ca_ (BK_Ca_) channels in smooth muscle cells of coronary arteries [72, 73]. Previous studies showed that H_2_S may augment the K_Ca_ channel current in vascular cells and activation of K_Ca_ channels is involved in H_2_S-induced vasodilatation [74, 75]. It would therefore be intriguing to unravel whether preserving K_Ca_ channel activity from ER stress takes part in H_2_S-conferred vascular protection against homocysteine, which may enrich our molecular-level understanding of the impact of H_2_S-homocysteine imbalance and the beneficial role of H_2_S in hyperhomocysteinemia.

## 9. H_2_S Antagonizes Homocysteine-Induced Vascular Injury: Other Potential Mechanisms?

As studies on the vascular effect of homocysteine keep advancing, more mechanistic linkage between H_2_S and homocysteine in the regulation of vascular function may be uncovered. For example, H_2_S is known to inhibit the angiotensin II type I receptor (Ang II/AT1R) pathway to regulate vascular function [76]. Recently, direct interaction and activation of AT1R by homocysteine were demonstrated to aggregate vascular injury [77]. Whether AT1R is a molecular basis underpinning vascular protection conferred by H_2_S against homocysteine therefore is a topic worthy of investigation. In addition, considering latest evidence regarding homocysteine-induced enhancement of T-type Ca^2+^ currents [78] and the inhibitory effect of H_2_S on CaV1.2 channels in vascular smooth muscle cells [79], modulation of voltage-dependent Ca^2+^ channels might also be a mechanism involved in H_2_S-mediated vascular protection in hyperhomocysteinemia although this warrants further investigation.

## 10. Homocysteine-Lowering Strategies and H_2_S Therapeutics in Treatment of Cardiovascular Disease

Despite the association between hyperhomocysteinemia and cardiovascular disease, randomized, placebo-controlled clinic trials evaluating the efficacy of homocysteine-lowering treatment, i.e., folic acid or/and B-vitamin supplementation, yielded inconsistent results; some favoring the effectiveness, e.g, slowing the progression of subclinical atherosclerosis and stroke, and improving endothelial function in coronary artery disease [80–83], while others showing no beneficial or only minor effect on the risk of major cardiovascular events in patients with vascular disease [84–86]. The causes leading to such contradictories remain not well understood, which might be related to the basal level of plasma folate, whether or not the patients on antiplatelet therapy, and the MTHFR C677T genotypes. Administration of folic acid and B-vitamin showed less effectiveness in lowering plasma homocysteine in subjects with normally high folate consumption before the treatment [87]. Suggestions have been made for clinical trials of homocysteine-lowering interventions via dietary supplementation with folic acid and B-vitamin to be conducted in regions where foods are not commonly fortified with folate [88]. In a randomized double-blind, placebo-controlled trial, Hankey and colleagues uncovered an interaction between antiplatelet therapy and the effect of folic acid/B-vitamin-based homocysteine-lowering therapy on major vascular events in patients with stroke or transient ischemic attack. They found that B-vitamins had no significant effect on the primary outcome in participants taking antiplatelet drugs at baseline whereas participants not taking antiplatelet drugs significantly benefited from the B-vitamin supplementation [89]. Recently, the China Stroke Primary Prevention Trial assessed individual variation in response to homocysteine-lowering interventions and suggested the effect modification by MTHFR polymorphisms. Compared with MTHFR677CC and CT genotypes, participants with the MTHFR677TT genotype exhibited a more prominent L-shaped curve between homocysteine and serum folate levels and required higher folate levels to eliminate the differences in homocysteine level by genotypes [90]. The influence of MTHFR C677T genotypes on the efficacy of folic acid and vitamin B12 in lowering homocysteine concentrations was also observed in hemodialysis patients [91]. A series of experiment performed in a mouse model containing a transgene (*Tg-I278T*), the most common mutation found in CBS-deficient patients, showed an entirely different response with regard to homocysteine-lowering diet as compared to the normal controls [92], which gives more support to the concept of gene-diet interaction in disease treatment. In addition, whether the newly found mutations of CBS in hyperhomocysteinemic patients, such as c.467T>C; p.Leu156Pro and c.808_810del; p.Glu270del, have impact on the therapeutic efficiency may need to be studied [93]. Taken together, well-constructed trials with consideration of the abovementioned factors are needed to provide conclusive answers to the clinical effectiveness of homocysteine-lowering strategies in reducing the incidence of cardiovascular complications.

As evidence concerning the safety and effectiveness of H_2_S-releasing therapeutics in animal models of cardiovascular disease keeps growing, attempts have been made to develop H_2_S-based drugs for human use, and the promise is now being demonstrated in clinical trials. In both spontaneously or two-kidney one-clip hypertensive rats, NaHS treatment significantly lowered the mean arterial pressure and improved vasodilatation [94, 95]. Activation of eNOS through PPAR*δ*/PI3K/Akt or PPAR*δ*/AMPK signaling [94] and restoration of NO bioavailability by decreasing the plasma level of the NOS inhibitor N^G^ monomethyl-l-arginine were revealed as underlying mechanisms [95]. Further studies of renal arteries from hypertensive patients and human umbilical vein endothelial cells subjected to angiotensin II exposure confirmed the protective effect of NaHS on endothelium and eNOS-NO functionality, supporting the potential of H_2_S-releasing drugs in the treatment of hypertension [94]. Nevertheless, clinical use of NaHS seems to be impractical due to its short half-life and toxicity, and novel H_2_S donors with enhanced efficacy and reduced toxicity are needed to realize H_2_S-based therapies. In a recent review article, Wallace and colleagues introduced the few H_2_S-releasing drugs that have progressed into clinical trials, such as H_2_S prodrug SG1002, an inorganic mixture (sodium polysulthionate) containing S_8_, Na_2_SO_4_, Na_2_S_2_O_3_, Na_2_S_3_O_6_, Na_2_S_4_O_6_, and Na_2_S_5_O_6_ for heart failure, and ATB-346, a nonsteroidal anti-inflammatory drug derived from naproxen but coupled to an H_2_S-releasing moiety for arthritis [96]. Alleviation of cardiac remodeling and afterload by SG1002 was recently proven in the CBS^+/-^ hyperhomocysteinemic mouse model [97]. By discussing the signaling pathways influenced by H_2_S-dependent sulfhydration that attenuates DNA damage, oxidative stress, and eNOS inhibition, Li et al. gave an overview of the development of H_2_S donors and the evolution of H_2_S therapeutics in cardiovascular disease with presenting the hypothesis that H_2_S may serve as a dual protector for both the heart and the kidney in cardiorenal syndrome [98]. The development of H_2_S-based therapy shall benefit from new techniques/materials that are able to control the amount of H_2_S released from the donor drug. The effectiveness of a synthesized peptide-based H_2_S-releasing hydrogel in reducing intimal hyperplasia of the vein grafts from patients undergoing bypass surgery is an example [99].

## 11. Summary and Future Perspectives

Homocysteine and H_2_S are both metabolites of sulfur-containing amino acids. Transsulfuration of homocysteine to cysteine is catalyzed by CBS and CSE, which are also key enzymes producing H_2_S from homocysteine and/or cysteine. Biogenesis of homocysteine and H_2_S is regulated by each other. H_2_S protects against homocysteine-induced vascular injury through multiple mechanisms including normalizing NO functionality by regulating eNOS signaling and alleviating oxidative stress and inflammation via restoration of redox balance, in which maintaining the structural and functional integrity of mitochondrion and ER plays a significant role. Recent findings of compromised ion channel activity in particular K_Ca_ channel activity in response to ER stress caused by homocysteine added new mechanistic insight into homocysteine-induced vascular injury and raised a topic worthy of investigation whether preserving ion channel activity by protecting ER takes part in H_2_S-conferred protection against homocysteine on vasculatures. Metabolic imbalance of homocysteine and H_2_S has been implicated in several types of cardiovascular disorders, which renders homocysteine-lowering and H_2_S-enhancing strategies promising therapeutics for cardiovascular disease. Furthermore, H_2_S/homocysteine ratio in comparison with homocysteine or H_2_S level alone may be a more reliable biomarker for cardiovascular-disease risk prediction. Clinical trials assessing the effectiveness of homocysteine-lowering interventions, i.e., folic acid or/and B-vitamin supplementation in reducing the incidence of cardiovascular complications should take into account the influence of dietary folate intake and antiplatelet treatment. Further consideration of MTHFR C677T and CBS genotypes is required for precision of homocysteine-lowering interventions in hyperhomocysteinemic individuals. Development of novel H_2_S-generating compounds with controlled-release properties and oral bioavailability is essential for clinical application of H_2_S therapies that are so far still at a very early stage. More importantly, development of drugs or interventions targeting the interplay between homocysteine and H_2_S to maintain the endogenous balance of these two molecules may hold even bigger promise for management of cardiovascular disorders.

## Abbreviations

MST: 3-Mercaptopyruvate sulfurtransferase
3-MP: 3-Mercaptopyruvate
K_Ca_ channels: Calcium-activated potassium channels
CVD: Cardiovascular disease
COPD: Chronic obstructive pulmonary disease
CBS: Cystathionine *β*-synthase
CSE: Cystathionine *γ*-lyase
CAT: Cysteine aminotransferase
ER: Endoplasmic reticulum
(ERO)-1*α*: ER oxidoreductase-1*α*
H_2_O_2_: Hydrogen peroxide
H_2_S: Hydrogen sulfide
MMPs: Matrix metalloproteinases
MS: Methionine synthase
MT: Methyltransferase
MTHFR: N-5,10-methylenetetrahydrofolate reductase
NO: Nitric oxide
NMDA-R1: N-methyl D-aspartate receptor
PDI: Protein disulfide isomerase
SAH hydrolase: S-Adenosylhomocysteine hydrolase
SAM synthetase: S-Adenosylmethionine synthetase
NaHS: Sodium hydrosulfide
SP1: Specificity protein-1
SOD: Superoxide dismutase
BH4: Tetrahydrobiopterin
THF: Tetrahydrofolate.
